# Switching and extension of transmission response, based on bending metamaterials

**DOI:** 10.1038/s41598-017-03824-4

**Published:** 2017-06-15

**Authors:** J. S. Hwang, Y. J. Kim, Y. J. Yoo, K. W. Kim, J. Y. Rhee, L. Y. Chen, Y. P. Lee

**Affiliations:** 1 Dept. of Physics, Hanyang University, Seoul, 04763 South Korea; 2 Dept. of Information Display, Sunmoon University, Asan, 336-708 South Korea; 3 Sungkyunkwan University, Suwon, South Korea; 4 Fudan University, Shanghai, China

## Abstract

The electromagnetically-induced transparency (EIT)-like effects in planar and non-planar metamaterials (MMs) were investigated for microwave (GHz) frequencies. The specific MMs used in this study consisted of a cut-wire resonator and a ring resonator, where were placed on the top and the bottom layers, respectively. A transmission window was produced, due to the interference between bright- and bright-mode coupling. Using the numerical and the experimental results, we demonstrate that the bending of MM leads to enhanced transmission and bandwidth, as well as an additional EIT-like peak. This provides an effective way of realizing the tunable devices, including the switching sensors.

## Introduction

Electromagnetically-induced transparency (EIT) is a quantum interference effect which is activated in coherent interaction between atomic ensembles^1^. The phenomenon gives rise to a sharp transmission window in the absorption spectrum. The EIT results in slow-light phenomena, this enhancing the nonlinearity associated with near-resonance effects and promoting a variety of applications, including nonlinear optics, quantum optical memories and slow-light devices^2–6^. However, the EIT phenomena in these atomic systems are hard to be realized experimentally owing both to the cryogenic operation temperatures, and the need for stable lasers to match the atomic transitions^7^. Recently, the coherent processes, leading to EIT, have been observed in MMs^8^. This can relax dramatically the difficulty in the experimental realization because of the room-temperature operation and of no need for external activation by pumping lasers. The MM structures reveal the EIT-like phenomena which are the result of Fano-type linear destructive interference among the artificial resonant elements^9^. The phenomena are obtained by the broken structural symmetry or by the near-field subwavelength-scale coupling^10–12^. The latter origin of EIT can be interpreted by the near-field coupling between a radiative bright resonator that couples strongly with the incident light, and a dark resonator that couples weakly with light^13, 14^. However, the EIT-like behavior also occurs without the dark-mode excitation, which is described by the interference between bright modes^15^. In addition, the EIT-like effect, based on the phase coupling, can be switched by adjusting the incident angle^16, 17^. Herein, we studied the EIT-like effect using bright- and bright-mode resonators^18^. Two types of resonators were fabricated at the top [a cut-wire resonator (CWR)] and the bottom [a ring resonator (RR)] of FR-4 substrate. By placing two types of resonators on the top and the bottom layers, they can have different phases. This allows us to have the classical analogue of EIT-like effect due to the phase coupling between CWR and RR, which leads to the enhanced transmission in the microwave range. To control effectively the transmission peak using two types of resonator modes, we have also investigated the bending effect at GHz frequencies in this EIT MM.

So far, only a few studies have been performed regarding this topic, which all reported simply on the planar substrates for the EIT-like effect working at GHz frequencies^19–21^. However, in our study here, we used non-planar substrate to elucidate the bending effect. The EIT-like feature could be controlled by adjusting the bending parameters of sample. This response tuning via bending the MM is useful for not only holographic and active filters, but applications involving biosensing^22–24^.

## Results and Discussion

**Figure 1:**
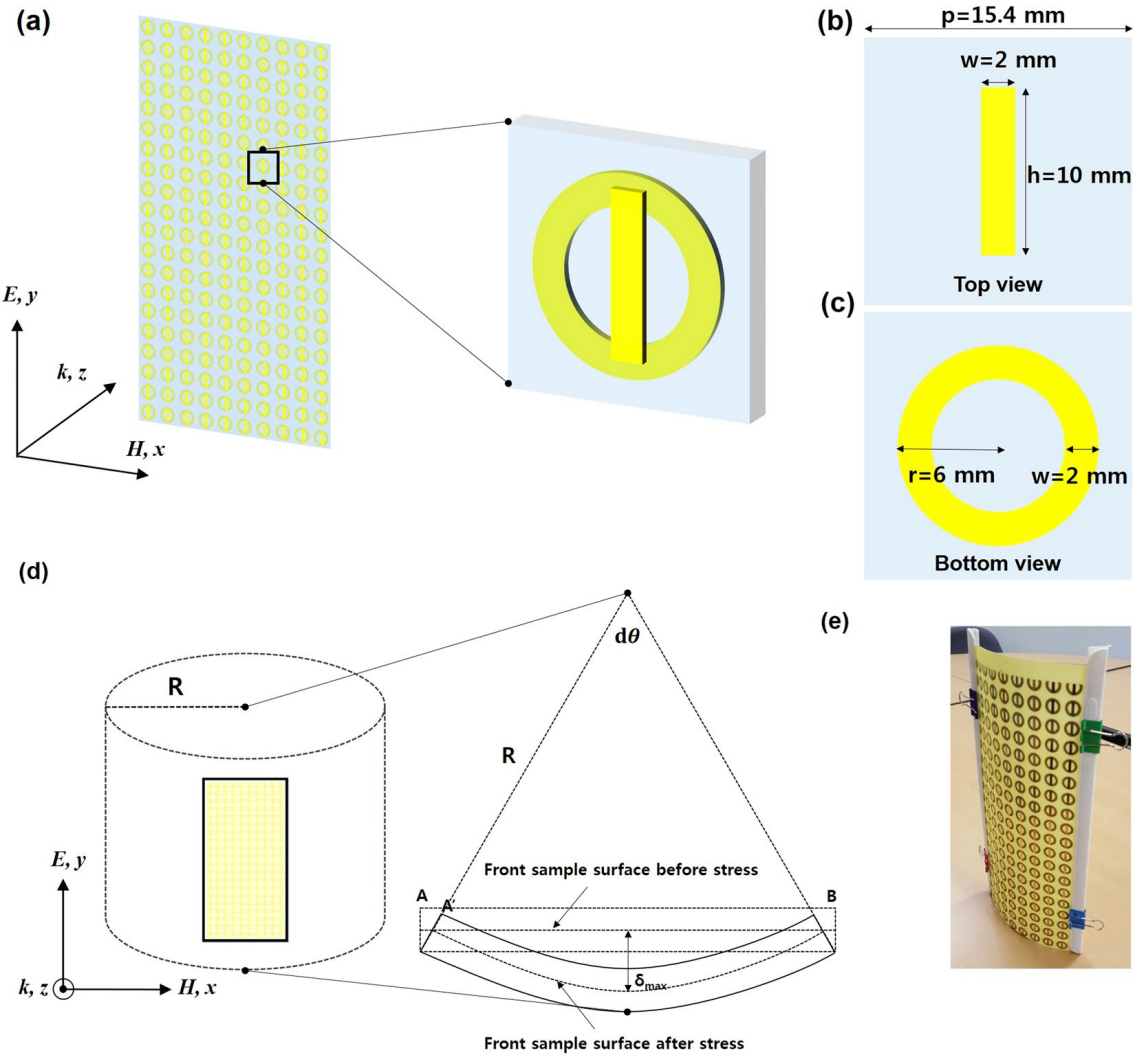
(**a**) Schematic view of the unit cell of proposed design. (**b**) Two-dimensional top view of a copper-based CWR and (**c**) bottom view of an RR with the geometrical parameters. (**d**) Schematic of the bent structure based on hollow tube. Polarization of incident wave is also shown. (**e**) Fabricated MM sample under strain.

The schematic diagram of the two types of resonators, which were fabricated at the top and the bottom layers, is presented in Fig. 1(a–c). The 0.035 mm-thick copper patterns with an electric conductivity of 5.8 × 10^7^ S/m were made on FR-4 substrate. The 0.4 mm-thick FR-4 dielectric substrate, which has a dielectric constant of 3.9 and a loss tangent of 0.025, is used as the spacer layer. We designed the structure which included CWR with the geometrical parameters of width (*w*) = 2 mm and height (*h*) = 10 mm, and RR whose geometrical parameters were optimized to have a radius (*r*) = 6 mm in order to achieve the same resonance frequency with CWR and RR. The periodicity of MM was set to be 15.4 mm. A hollow tube with a radius *R* was used to simulate the transmission properties of bent structure, and we have fabricated a sample as shown in Fig. 1(d) and (e) to investigate the experimental transmission.

**Figure 2:**
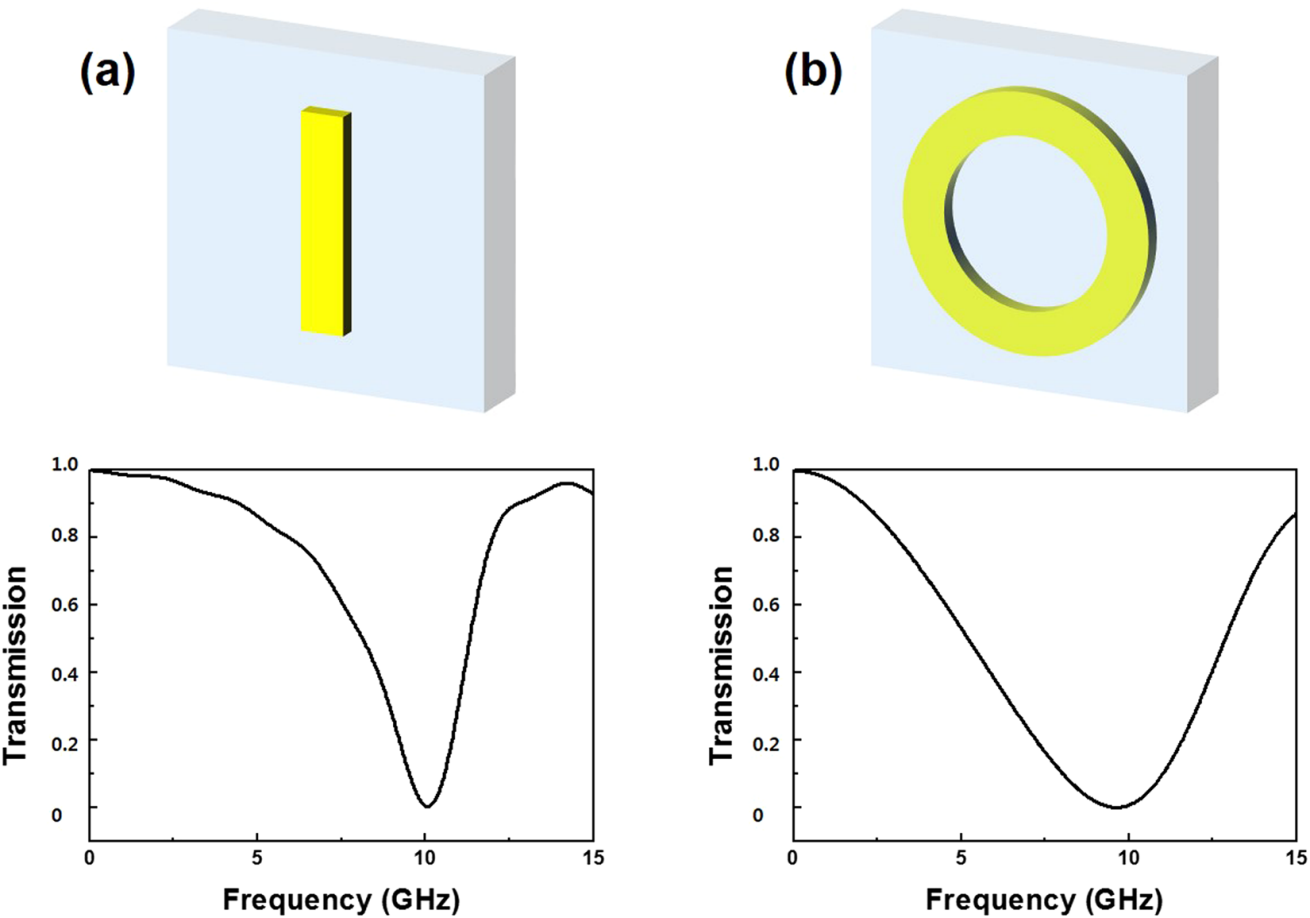
Simulated spectra of the transmission at the normal incidence for (**a**) CWR alone (**b**) RR alone.

**Figure 3:**
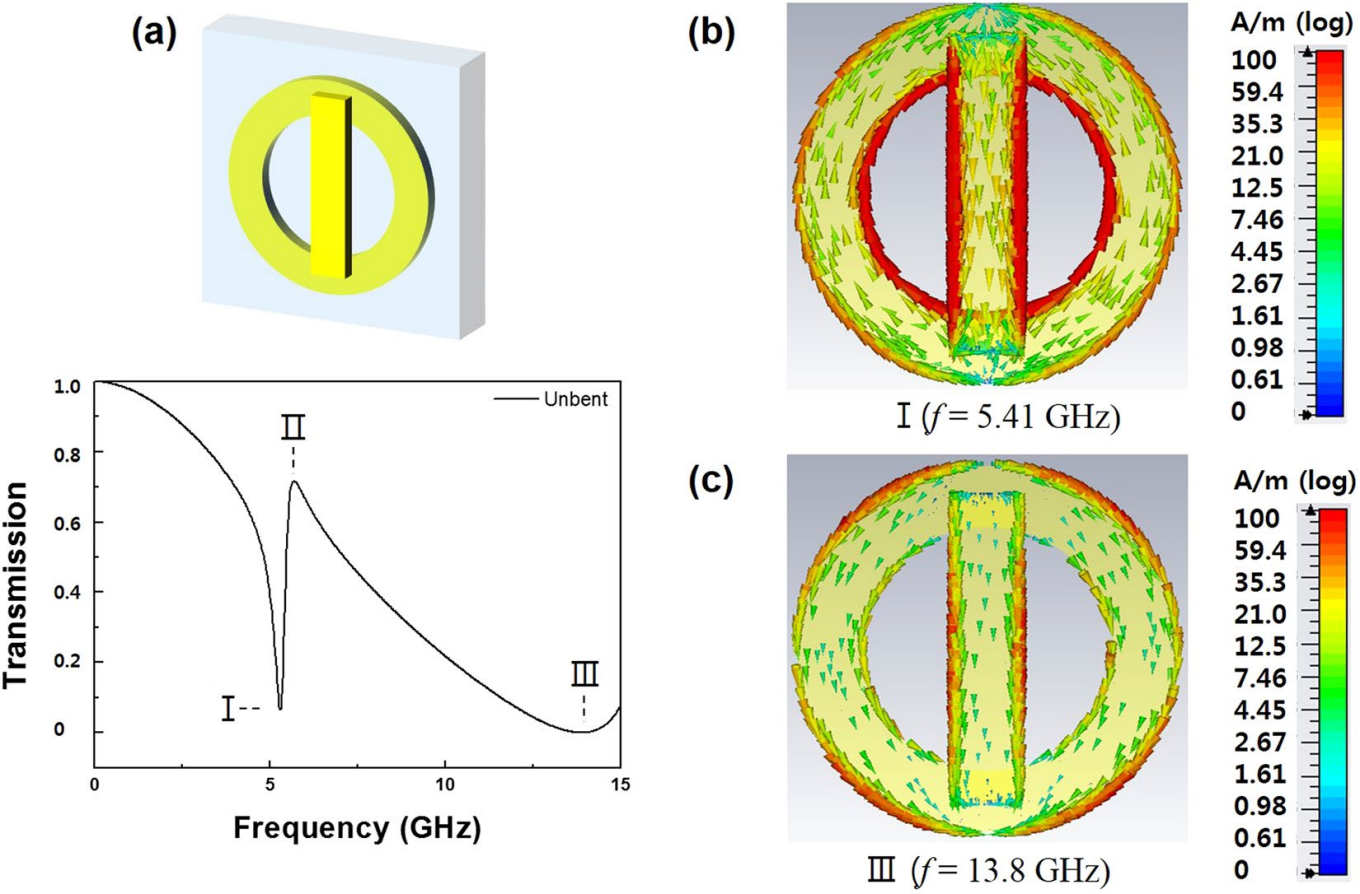
(**a**) Simulated spectra of the transmission at the normal incidence for the CWR-RR structure. Surface-current distributions at (**b**) 5.41 and (**c**) 13.8 GHz.

Figure 2(a) and (b) present the simulated transmission spectra of CWR alone and RR alone, respectively. The incident wave was polarized along the *y* direction, directly exciting the CWR and the RR. The Q-factor is evaluated by using $Q={\omega }_{0}/{\rm{\Delta }}{\omega }_{FWHM}$, where ${\omega }_{0}$ is the resonance frequency and ${\rm{\Delta }}{\omega }_{FWHM}$ is the full width at half maximum. The fundamental mode of CWR shows a low radiative loss, a narrow bandwidth and a high Q (${{\rm{Q}}}_{1}=3.53$). On the other hand, the RR presents a broad bandwidth and a low Q (Q_2_ = 1.39). The CWR exhibits a resonance at 10.1 GHz, which is higher by 0.309 GHz than that of the RR. Interestingly, when the CWR and the RR are combined as in Fig. 3(a), the interference between two eigenmodes leads to a transmission window (II) lying between two well-pronounced transmission dips (I and III). This effect is well-known to be the EIT-like effect, which originates from the bright- and bright-mode coupling. A destructive interference occurs, due to the bright- and bright-mode coupling, and this interference makes the opaque medium transparent. Each resonator exhibits resonance near 10 GHz while the transmission window appears at 5.69 GHz. To confirm this fact, we simulated the distribution of surface currents occurring in the transmission-dip region. As shown in Fig. 3(b) and (c), the surface current is formed strongly in antiparallel way for transmission dip I, but that is formed in parallel way for dip III. In the transmission dip I, a trap mode is shown, based on the magnetic resonance, which produces high Q resonance. Conversely, in transmission dip III, the radiation mode, based on the electric-resonance phenomenon, induces a broad resonance spectrum, which results in the transmission to appear at 5.69 GHz instead of 10.1 GHz.

**Figure 4:**
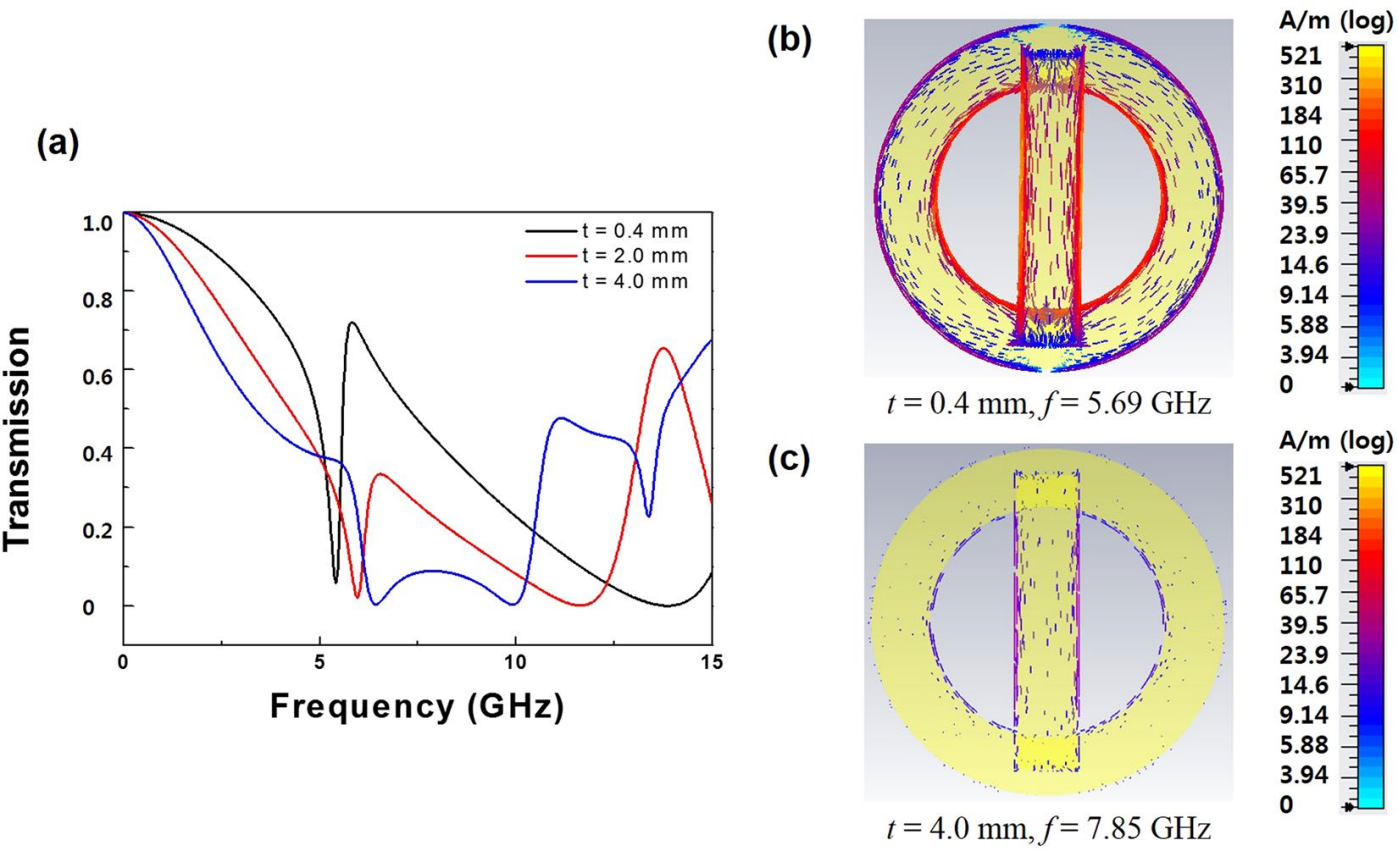
(**a**) Simulated spectra of the transmission at the normal incidence for the CWR-RR structure by varying the thickness of FR4 substrate. Surface-current distributions at (**b**) *t* = 0.4 mm and 5.69 GHz, and (**c**) *t* = 4.0 mm and 7.85 GHz.

The manifestation of EIT phenomenon depends on the difference in the phase between the two resonators in our structure. The simulated transmission spectra for MM by varying the thickness of FR4 substrate are shown in Fig. 4(a). As the thickness *t*_*s*_ of FR4 increases, the transmission at 5.69 GHz decreases significantly. Obviously, varying the distance between the two resonators can further weaken the phase coupling strength between CWR and RR. The surface-current distribution for *t*_*s*_ = 0.4 mm and *f* = 5.69 GHz is shown in Fig. 4(b), where a strong current is generated in the CWR and the RR. On the other hand, for *t*_*s*_ = 4.0 mm, a weaker current distribution is observed at the CWR and the RR at 7.85 GHz, as shown in Fig. 4(c). Thus, the coupling between CWR and RR still occurs but is weakened.

We can describe the coupling effect of EIT-like transparency using the Lorentz oscillator model. In the following coupled differential equations, it is considered that both oscillators are strongly coupled to the incoming electric field $E(t)={E}_{0}{e}^{-iwt}$.

1 $$\frac{{\partial }^{2}}{\,\partial {t}^{2}}{x}_{1b}({\rm{t}})+{\gamma }_{1b}\frac{\partial }{\partial {\rm{t}}}{x}_{1b}({\rm{t}})+{\omega }_{1b\,}^{2}{x}_{1b}({\rm{t}})+{k}^{2}{x}_{2b}({\rm{t}})={E}_{1b}({\rm{t}}),$$

2 $$\frac{{\partial }^{2}}{\partial {t}^{2}}{x}_{2b}({\rm{t}})+{\gamma }_{2b}\frac{\partial }{\partial {\rm{t}}}{x}_{2b}({\rm{t}})+{\omega }_{2b\,}^{2}{x}_{2b}({\rm{t}})+{k}^{2}{x}_{1b}({\rm{t}})={E}_{2b}({\rm{t}}).$$

These coupled equations show that the relative coupling of incoming radiation with the two bright modes. Here, (${x}_{1b}$, ${x}_{2b}$), (${\omega }_{1b}$, ${\omega }_{2b}$), and (${\gamma }_{1b}$, ${\gamma }_{2b}$) are the displacements, the resonance frequencies and the damping rates of two bright modes, respectively, and (${E}_{1b,}\,{E}_{2b,}$) are assumed to be proportional to the electric field *E*_*0*_ of electromagnetic wave with proportionality constants of (${\alpha }_{1b},\,{\alpha }_{2b}$). *k* defines the coupling coefficient between two bright modes. We solve the equations by putting ${x}_{1b}(t)={N}_{1b}{e}^{-iwt}$ and ${x}_{2b}(t)={N}_{2b}{e}^{-iwt}$, where *N* is constant.

3 $$[\begin{array}{c}{x}_{1b}\\ {x}_{2b}\end{array}]=\frac{1}{({\omega }^{2}-{\omega }_{1b}^{2}+i\omega {\gamma }_{1b})({\omega }^{2}-{\omega }_{2b}^{2}+i\omega {\gamma }_{2b})-{k}^{4}}\times [\begin{array}{c}({\omega }^{2}-{\omega }_{2b}^{2}+i\omega {\gamma }_{2b})({E}_{1b})+{k}^{2}({E}_{2b})\\ ({\omega }^{2}-{\omega }_{1b}^{2}+i\omega {\gamma }_{1b})({E}_{2b})+{k}^{2}({E}_{1b})\end{array}].$$

The values of damping rates ${\gamma }_{1b}$ and ${\gamma }_{2b}$ are calculated to be 2.85 and 7.01 GHz, respectively, which are obtained from the linewidths of the curves in Fig. 2(a) and (b). The coupling coefficient *k* for transmission spectrum is calculated by using the formula given in refs 25 and 26, which are derived from Eq. 3. We obtained the *k* value at 5.65 GHz for the CWR-RR structure, resulting from the corresponding transmission peak. We can predict the EIT-like phenomena, based on the coupling between CWR and RR through this classic model.

**Figure 5:**
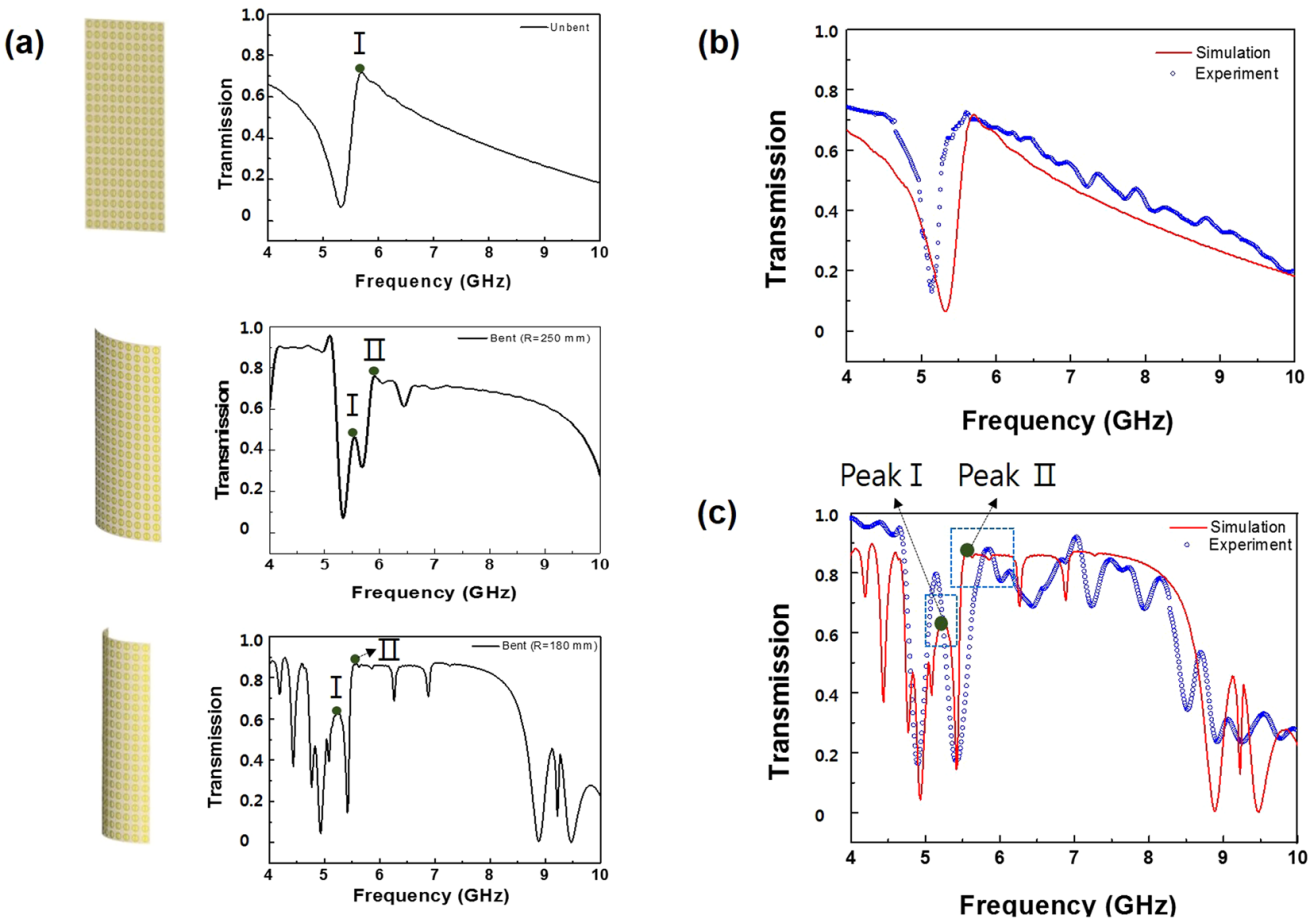
(**a**) Simulated transmission spectra of the MM for different bending strains (R = 250 and 180 mm). Transmission spectra of the CWR-RR structure for the (**b**) unbent and (**c**) the bent state (R = 180 mm).

**Figure 6:**
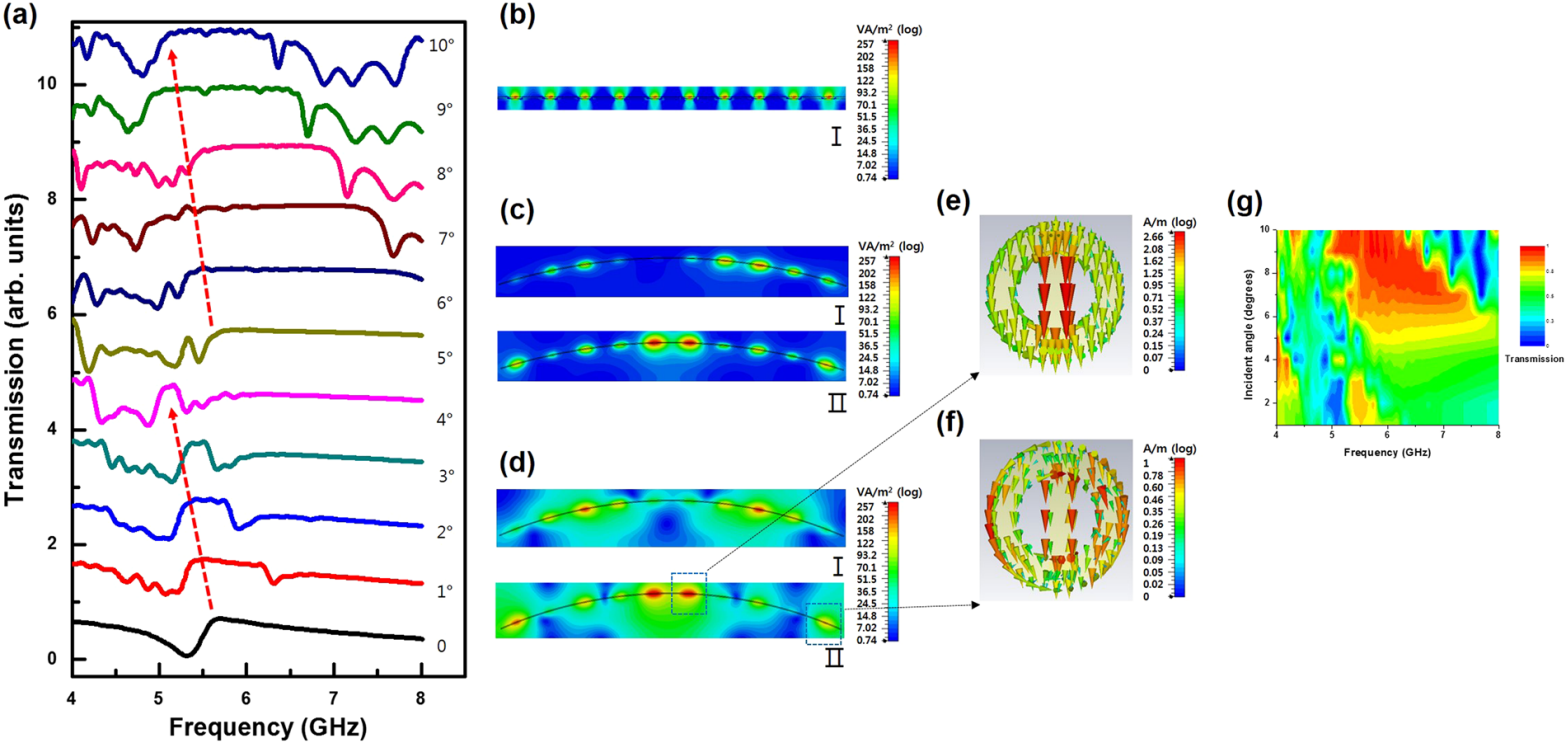
(**a**) Simulated spectra of the transmission for incident angle from 0 to 10 degrees in step of 1 degree for the unbent sample. Power-loss-density distribution (**b**) at 5.69 GHz (I) in the unbent state (**c**) at 5.55 (I) and 5.91 (II) GHz in the bent state (R = 250 mm), and (**d**) at 5.26 (I) and 5.69 (II) GHz in the bent state (R = 180 mm). Surface current distributions corresponding to transmission peak II at incident angle of (**e**) 0 and (**f**) 10 degrees. (**g**) Dispersion plot of the transmission as a function of frequency and incident angle for the CWR-RR structure.

To elucidate the EIT-like effect in the bending system, a hollow tube with a radius of 250 mm and a radius of 180 mm was used to simulate a bending strain to the sample. As shown in Fig. 5(a), we detected an additional transmission peak of 47% at a frequency of 5.55 GHz for a radius of 250 mm and the transmission was improved to be 63.3% at 5.26 GHz for a radius of 180 mm. Besides, the wide-band transmission is observed in a range of 5.58 to 8.23 GHz. We also found a significant increase in the transmission to be between 78.1% and 87.3% in the wide band of 5.58–8.23 GHz after bending, with respect to the transmission between 33.7 and 71.8% before bending. The comparison of measured and simulated transmission spectra is demonstrated in Fig. 5(b) and (c) for both states. The well-defined EIT-like peak was experimentally observed, and it was in a fairly good agreement with the simulated transmission spectrum. The real permittivity of dielectric substrate might differ from that in the simulation, which leads to the slight shift of resonance frequency between simulation and experiment. To check further the CWR-RR transmission vs. bending degree, we calculated the transmission spectrum of a single unit cell with periodic boundary conditions for different angles of incidence, which was investigated for oblique incidence through a parametric sweep on the incident angle *θ* from 0 to 10 degrees with step of 1 degree, as given in Fig. 6(a). As observed, the CWR-RR transmission peak progressively shifts from 5.58 to 5.16 GHz as the angle of incidence increases from 0 to 4 degrees. At an incident angle of 5 degrees, another transmission peak appears and, as the incident angle increases from 5 to 9 degrees, it gradually moves from 5.75 to 4.98 GHz. In order to elucidate the influence of angle of incidence on transmission peak I and II, the power-loss-density distribution was further simulated. That of the unbent sample is shown in Fig. 6(b) and the power-loss-density distributions of the sample with a hollow tube radius of 250 and 180 mm are in Fig. 6(c) and (d), respectively. For the unbent sample, that at 5.69 GHz was simulated. It is confirmed that the excitation occurs at the normal incidence. When bending was performed like a hollow tube with a radius of 250 mm, in the case of transmission peak I, the excitation occurred at an incident angle of 5 degrees. On the other hand, for transmission peak II, the excitation occurred at the normal incidence, as shown in Fig. 6(c). In Fig. 6(d), the case of a radius of 180 mm, for transmission peak I, the excitation of surface electromagnetic wave occurs at an incident angle of 6 degrees and the transmission is due to the destructive interference between two resonators at this incident angle. For transmission peak II, the excitation of surface electromagnetic wave is induced at incident angles of 0 and 10 degrees. Transmission peak II occurs owing to the destructive interference between two resonators at these incident angles. Figure 6(e) and (f) show the surface-current distributions corresponding to transmission peak II at incident angles 0 and 10 degrees, respectively. The parallel current flows between CWR and RR, and thus the total magnetic moment in the *x* direction is cancel out. That is, scattering is suppressed due to the destructive interference between radiation losses from two resonators. When the incident angle is 0, strong excitation occurs in the CWR as compared with the RR. However, when the incident angle is 10 degrees, a strong current is generated not only at the CWR but also at the edge of RR, as shown in Fig. 6(f). Hence, the EIT-like effect is markedly enhanced by the superposition of two states which are excited at 0 and 10 degrees. As shown in Fig. 5(b) and (c), due to the superposition, the transmission is improved from 71.8% to 87.3% [in Fig. 5(c)] at 5.58 GHz. Figure 6(g) presents the dispersion plot of transmission response of the CWR-RR as a function of frequency and angle of incidence. A planar MM exhibits an angular dependence of transmission. When the incident angle is larger than 6 degrees, the maximum transmission comes to be 87% in a range of 5.08–6.45 GHz. It is anticipated that the EIT-like effects by two types of resonator modes can be manipulated by changing the incident angle, which can be employed for the transmission switching.

**Figure 7:**
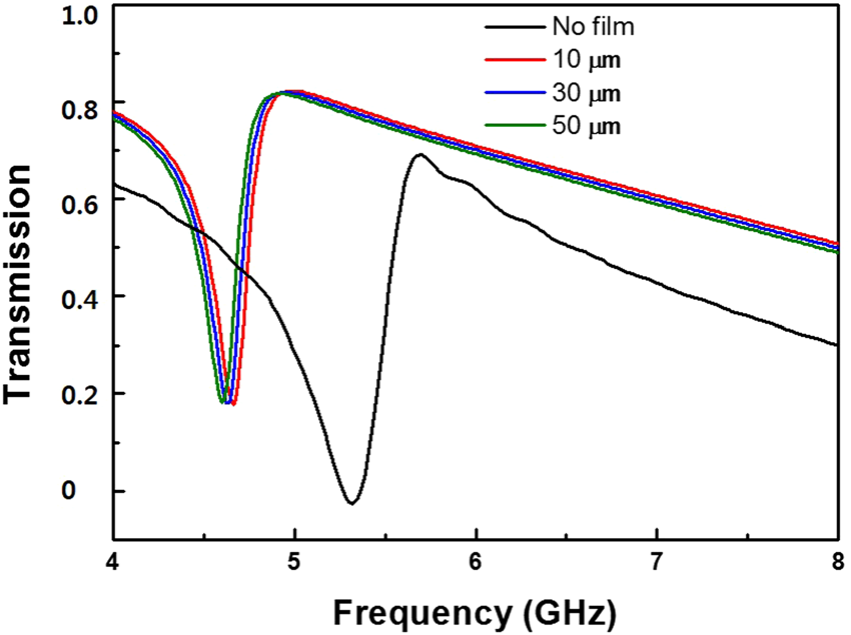
Simulated shift of the resonance frequency of the EIT-like MM by increasing the thickness of overlayer.

We also investigated the capability of CWR-RR structure for the thin-film sensor, especially, in biological and biochemical fields. In order to analyze the sensitivity, the uniform dielectric overlayers were deposited on the CWR plane and the shift of resonance frequency was studied. The following three different thicknesses were performed: the CWR-RR with a overlayer of FR-4 with thickness of 10, 30 and 50 μm, as in Fig. 7. In the absence of overlayer, the resonance frequency was detected at 5.31 GHz. When the thickness of overlayer increases, the resonance frequency shifts continuously to lower frequency. The maximum shift of 711 MHz was observed for 50-μm thickness of FR-4, corresponding to a shift of 13.4% with respect to the case without overlayer. The sensitivity is defined as equation of *∆f*/(*nt*), in which *∆f* is the resonance-frequency shift, *n* is the refractive index, and *t* is the thickness^27^. Consequently, the sensitivity of CWR-RR is calculated as 7.2 MHz/μm. The proposed structure can achieve a larger resonance-frequency shift than the case of split-ring resonator^28^.

## Conclusions

This study reports the investigation of EIT-like effects at microwave frequencies, by utilizing a CWR-RR structure. We were able to implement successfully that the EIT-like effects are realized in the bilayer structure through the bright- and bright-mode coupling. It is confirmed that the transmission is manipulated by the bending parameters. In particular, at the bent state, the additional transmission peak and the wide-band transmission were observed. Our approach provides significant advance in tuning the electromagnetic response, which is useful for the potential switching sensors. In addition, our design concept might be beneficial in paving the way to thin-film sensors which are highly sensitive for biological and biochemical applications.

## Method Summary

### Electromagnetic-wave simulations

To obtain the scattering parameters of supercell, the non-periodic boundary conditions was employed in the finite-difference time-domain method by using CST Microwave Studio. For the simulation, in the tetrahedral mesh, the boundary conditions of perfect electric and magnetic conductor were assigned for the side walls (*x*–*y* and *x–z* planes) of the 10 × 20 supercell, and the supercell was placed between two waveguide ports. The boundary along *z*-direction is set as open at a distance of 150 cm from the structure.

### Electromagnetic-wave transmission measurement

The transmission spectra were measured in an anechoic chamber. A Hewlett Packard E8362B network analyzer was employed, which was connected to microwave standard-gain horn antennas. The distance between two antennas was kept at 150 cm for all the measurements to minimize the near-field effects.
